# Inflammatory Biomarkers, Hematopoietic Stem Cells, and Symptoms in Breast Cancer Patients Undergoing Adjuvant Radiation Therapy

**DOI:** 10.1093/jncics/pkaa037

**Published:** 2020-05-08

**Authors:** Wei Shi, Shagun Misra, Madeline Li, Jie Su, Lisa P Chong, Megan McCuske, Justin Williams, Wei Xu, Laleh S Ghoraie, D Robert Sutherland, Kathy Han, Mark D Minden, Scott V Bratman, Kenneth W Yip, Fei-Fei Liu

**Affiliations:** p1 Princess Margaret Cancer Centre, University Health Network, Toronto, Ontario, Canada; p2 Radiation Medicine Program, Princess Margaret Cancer Centre, University Health Network, Toronto, Ontario, Canada; p3 Department of Radiation Oncology, University of Toronto, Toronto, Ontario, Canada; p4 Department of Supportive Care, Princess Margaret Cancer Centre, University Health Network, Toronto, Canada; p5 Department of Psychiatry, University of Toronto, Toronto, Ontario, Canada; p6 Division of Biostatistics, Princess Margaret Cancer Centre, University Health Network, Toronto, Ontario, Canada; p7 Department of Biostatistics, University of Toronto, Toronto, Ontario, Canada; p8 Department of Hematology, Princess Margaret Cancer Centre, University Health Network, Toronto, Ontario, Canada; p9 Department of Medical Biophysics, Faculty of Medicine, University of Toronto, Toronto, Ontario, Canada

## Abstract

**Background:**

Fatigue and insomnia are common symptoms experienced by breast cancer patients undergoing adjuvant radiation therapy (RT), yet the underlying mechanisms of these symptoms are unclear. In particular, the roles of hematopoietic stem cells (HSCs) and inflammatory cytokines remain to be elucidated.

**Methods:**

Breast cancer patients (n = 147) completed questionnaires to longitudinally assess symptoms before, during, and after adjuvant RT. Phlebotomies were performed prior to RT, at the second and fifth treatment fractions, end of treatment (EOT), and 1 month after completing RT, assessing for CD34^+^, CD45^+^, full hematology, and 17 inflammatory cytokines. The associations between symptoms and all biomarkers were evaluated. All statistical tests were 2-sided.

**Results:**

General fatigue and insomnia worsened with RT, with peak levels observed at EOT, which remained statistically significant even after controlling for anxiety and depression (*P* < .05 for all). CD34^+^, CD45^+^, white blood cell, and lymphocyte counts decreased, with the lowest levels also observed at EOT (*P* < .001). Fatigue and insomnia were associated with changes in both interferon γ-induced protein 10 (IP-10) - (*P* = .03 and *P* = .01, respectively) and tumor necrosis factor receptor II (TNF-RII) (*P* = .02 and *P* = .006, respectively), while mental fatigue was associated with increased matrix metalloproteinases-2 (MMP-2) levels (*P* = .03). Patients who received prior chemotherapy demonstrated statistically significantly greater severity in all symptoms, with lower baseline HSC levels.

**Conclusions:**

This is the first longitudinal study to examine linkages between symptoms, HSCs, and cytokines, demonstrating that fatigue and insomnia shared associations with increasing serum levels of IP-10 and TNF-RII, and mental fatigue was associated with increasing serum levels of MMP-2. Our findings highlight opportunities for further research into mechanisms and potential interventions for these symptoms.

One of the key adjuvant modalities used in breast cancer management is radiotherapy, which is delivered locally to the breast plus or minus regional lymph nodes. Common and disabling side effects of radiation therapy (RT) include symptoms such as fatigue, insomnia, and depression, which can adversely affect overall quality of life (1,2). Evidence suggests that approximately 30%–60% of cancer patients report fatigue of varying severity (3), with sleep disturbance and depression ranging from 30% to 70% (4) and 14% to 27% (5), respectively. In cross-sectional studies, approximately one-third of breast cancer (BC) survivors experience persistent fatigue before, during, and after cancer therapy, which may last for many years (3). These data emphasize the need for investigations into causative mechanisms to develop either prevention or mitigating strategies (6).

Fatigue is a complex symptom that can be confounded by many parameters, including anxiety, depression, and insomnia. These symptoms frequently co-occur in cancer patients, suggesting a common underlying mechanism. Anemia, hypothalamic dysregulation, and alterations in muscle metabolism have been shown to be contributing factors (7). Pro-inflammatory cytokines released by tumors (8), and induced by cancer treatments such as RT and chemotherapy (9), may affect signaling pathways in the central nervous system (CNS), triggering fatigue or other symptoms (10). Furthermore, the host inflammatory response may persist after treatment completion, leading to alterations in homeostasis (3). Associations between fatigue and serum levels of cytokines such as interleukin 1 beta (IL-1β), tumor necrosis factor alpha (TNF-α), and C reactive protein (CRP) have been reported in BC patients (11), although many studies report inconsistent findings (12). Many cytokines (eg, IL-1β, IL-6, and TNF-α) are also involved in regulating hematopoietic stem cell (HSC) functions, including self-renewal, differentiation, and maintenance of homeostasis of the hematopoietic system (13). In addition, radiation can induce HSC apoptosis and senescence and also cause damage to the HSC niche (14). CD34, a cell surface marker, is commonly used to identify HSCs, and CD45 is a hematopoietic lineage-restricted antigen that is expressed on all hematopoietic cells (15). CD45 dephosphorylates and degrades the C-X-C motif chemokine ligand 12C-X-C motif chemokine ligand 12 or stromal cell-derived factor 1 alpha (CXCL12 or SDF-1α) complex, resulting in HSC mobilization from the bone marrow into the blood stream (16). To our knowledge however, there is no direct evidence linking HSCs with these symptoms. Our previous study demonstrated that local RT altered several cytokines that affect HSC trafficking from the bone marrow, inducing the homing of HSCs to the local site of RT (17). A follow-up study in BC patients subsequently revealed that CD34^+^ HSCs in circulation declined over the first week of RT (18).

In the current study, we hypothesized that local BC RT induces several different cytokines that can mediate both HSC trafficking and symptoms such as fatigue and insomnia. The changes in fatigue, insomnia, depression, and anxiety during the course of RT were assessed. Specific cytokines and circulating HSCs that correlated with the development of these complex symptoms were identified.

## Materials and Methods

### Patient Population and Procedures

This longitudinal cohort study was conducted at the Princess Margaret Cancer Centre, University Health Network. Between February 2010 and July 2014, 151 BC patients with primary tumor resections who met the eligibility criteria (aged 18 years and older, histologically proven ductal carcinoma in situ, or invasive ductal or lobular carcinoma, with or without chemotherapy) were accrued to this study. Adjuvant chemotherapy patients were recruited 4 to 6 weeks after chemotherapy completion. Patients with previous bone marrow transplant and/or preexisting hematologic conditions, such as myelodysplasia or chronic leukemia, were excluded.

The first 34 patients underwent phlebotomies for CD34^+^, CD45^+^ and full hematological analysis prior to commencing breast RT (D1), at the second (D2) and fifth treatment fractions (D5), and 1 month after completion of RT (1 M). Based on the results of initial assessments, the remaining 113 patients were scheduled to undergo phlebotomy at the end of treatment (EOT), in addition to phlebotomies at D1, D2, D5, and 1 M and full hematological analysis. Serum samples were collected at the same time points for inflammatory cytokine analyses. For each patient, phlebotomies were performed within the same 4-hour time period to minimize diurnal variations (19). All participants provided written informed consent, and the study was approved by the Research Ethics Board of the University Health Network.

### Study Questionnaires

Evaluations of patient-reported outcomes were conducted using standardized self-assessment questionnaires during the same study time points. Fatigue was assessed using the Multidimensional Fatigue Inventory, which consists of 20 items, including 5 subscales of 4 items each: general fatigue, physical fatigue, reduced activity, reduced motivation, and mental fatigue (range 4–20) (20,21). Anxiety and depression were assessed using the Hospital Anxiety and Depression Scale (22), a 14-item self-reported measure with separate 7-item subscales. It was specifically designed for use by the medically ill; the depression subscale places emphasis on anhedonia and does not include somatic items. Each subscale is scored from 0 to 21, where cutoff scores of 8 are most widely used to indicate probable anxiety or depression (23). Insomnia was evaluated using the Insomnia Severity Index, a 7-item scale that has been validated in cancer populations. Sum scores range from 0 to 28, with a cutoff score of 8 indicating clinically significant insomnia (24).

### HSC Measurements

CD34^+^ and CD45^+^ cells were quantified using flow cytometry. Complete blood counts were obtained using standard clinical hematology laboratory practices. Absolute viable CD34^+^ and CD45^+^ cells were quantified using single-platform International Society of Hematotherapy and Graft Engineering methodology (25). Methodological details are provided in the Supplementary Materials and Methods section (available online).

### Serum Inflammatory Marker Assays

Supplementary Table 1 (available online) outlines all the blood tests that were performed, including 17 inflammatory pathway biomarkers, consisting of CRP, interferon gamma (IFN-r), interferon-alpha 2a (IFN-α2a), interleukin-10 (IL-10), interleukin-17A (IL-17A), IL-1β, interleukin-1 receptor antagonist (IL-1ra), (interleukin-4 (IL-4), interleukin-6 (IL-6), interferon γ-induced protein 10 (IP-10), monocyte chemoattractant protein-1 (MCP-1), matrix metalloproteinases-2 (MMP-2), matrix metalloproteinases-9 (MMP-9), SDF-1a, transforming growth factor beta 1 (TGF-β1), tumor necrosis factor receptor II (TNF-RII), and TNF-α. These cytokines were quantified using custom multiplexed electrochemiluminescence immunoassays (Meso Scale Discovery, Gaithersburg, MD). Further technical details are provided in the Supplementary Materials and Methods section (available online).

### Statistical Analyses

Descriptive statistics (number, minimum, maximum, mean, and standard deviation) were used to characterize fatigue scores, measurements of cytokines, and other circulating biomarkers. The mixed effects model was used for a trend test on the longitudinal variables, including linear and quadratic effects, as well as within-person associations between changes in biomarkers and symptoms. The variance components structure was applied to the model. Random intercept and random slope have been incorporated into the model. The linear mixed model was used to assess the association between inflammatory markers and fatigue, as well as other symptoms, taking into account the repeated measure within subjects over time. This model was also used to compare fatigue scores, cytokines, and other biomarkers with clinical factors such as age, type of therapy, menopausal status, RT dose, radiation fields, and days of RT. Two-sided tests with a statistical significance level (α = 0.05) were used. Analyses were performed using SAS 9.4 (SAS Institute, Cary, NC) and R 3.1.2 (R Core Team, Vienna, Austria). Principal Component Analysis was conducted on the fatigue scores, which identified the top major principal components (PCs). The correlation between top PCs, cytokines, and other biomarkers were further explored using the same statistical analysis tools as with the single fatigue scores.

## Results

### Patient Characteristics

Of the patients, 151 completed all requested questionnaires, with blood samples collected on 147 patients. Among the 147 patients, 113 completed all 5 time points for HSC and cytokine analysis, and 34 provided samples for D1, D2, D5, and 1 M time points according to the initial schedule of assessments.

The clinical characteristics of the 147 patients are presented in Table 1. The median age was 56 years, with the majority being postmenopausal (79 of 147 [53.7%]), with early stage Tis/T1 disease (104 of 147 [70.7%]). Hypofractionated radiation (42.4 Gy/16 fractions) was delivered to 36.7% (54 of 147) of patients, and conventional fractionated radiation was delivered to all other patients. The majority were treated using a 2-field technique (112 of 147 [76.2%]). More than one-third of patients (52 of 147 [35.4%]) were treated with adjuvant chemotherapy before starting radiation therapy, and 62.6% (92 of 147) were being treated with adjuvant hormone therapy.

**Table 1.** Clinical characteristics of patients (n = 147)

Characteristic	No. (%)
Median age, y	56
Range	32–83
Menopausal status	
Pre	48 (32.6)
During	16 (11.0)
Post	79 (53.7)
Unknown	4 (2.7)
Surgery	
Lumpectomy	131 (89.1)
Mastectomy	13 (8.9)
Modified radical mastectomy	3 (2.0)
T stage	
Tis/T1	104 (70.7)
T2	36 (24.5)
T3	7 (4.8)
Radiation fields	
2F	112 (76.2)
3F	9 (6.1)
4F	24 (16.3)
Unknown	2 (1.4)
Radiation therapy dose	
42.4 Gy/16f	54 (36.7)
50∼60 Gy/21∼25f	93 (63.3)
Adjuvant chemotherapy	
Yes	52 (35.4)
No	95 (64.6)
Hormone therapy	
Yes	92 (62.6)
No	54 (36.7)
Unknown	1 (0.7)

### Changes in Symptoms During and Post-RT

**Figure 1.**
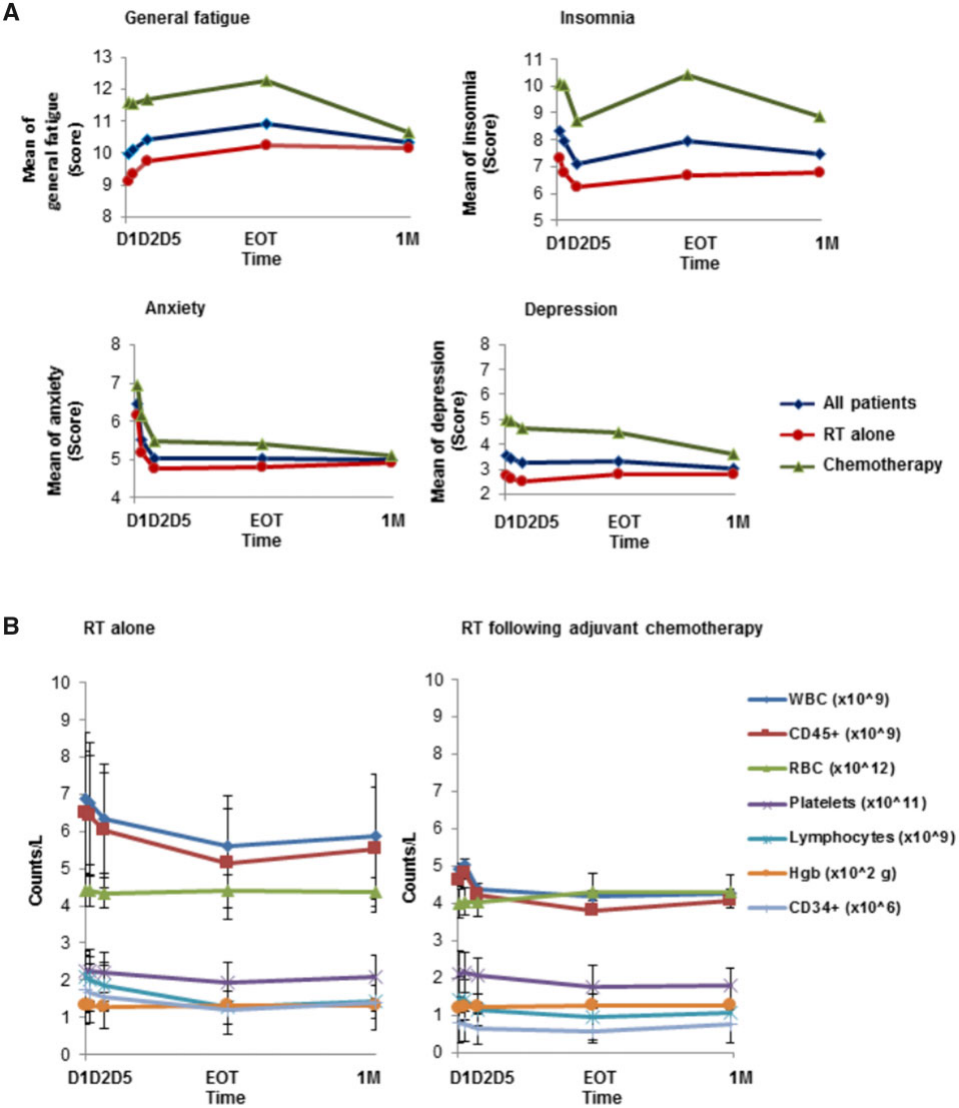
Changes in symptoms and hematologic biomarkers. **A**) Symptom changes during and after radiation therapy (RT). Mean severity levels of component scores in general fatigue; insomnia; anxiety; and depression, in patients during and 1-month post-RT. Time on the x-axis denotes first phlebotomy (D1, pretreatment), second (D2) and fifth treatment fraction (D5), and final day of RT (end of treatment [EOT]), and 1 month post-RT (1 M). All data are represented as mean of the absolute count for the entire cohort, for each time point. **B**) CD34+ (HSCs) and other biomarker changes during and after RT. Counts of WBCs, CD45+ cells, RBCs, platelets, lymphocytes, Hgb, and CD34+ cells are plotted as a function of RT alone or RT following adjuvant chemotherapy. Patients receiving chemotherapy had lower baseline levels of CD34+, CD45+, WBCs, and lymphocytes that were statistically significant compared to RT only, whereas the average baseline counts of RBCs, Hgb, and platelets were similar across the entire cohort. All data are presented as mean + SD. Hgb = hemoglobin; RBC = red blood cell; WBC = white blood cell.

The mixed effects model was used for a longitudinal trend test. As shown in Figure 1A, general fatigue increased during RT, with peak fatigue reported at EOT, wherein the mean change in worsening general fatigue from D1 to EOT was statistically significant (Table 2; *P* < .001). Insomnia initially improved during the first week of RT, but then worsened toward EOT (Figure 1A and Table 2; *P* = .004). Worsening of general fatigue was persistent even after adjusting for anxiety, depression, and insomnia. Consistent with previous studies (9,26), this difference was not affected by patient age (≤60 vs >60 years), menopausal status (pre-/peri- vs post-), radiation dose and fields, or hormone therapy. Patients who received prior chemotherapy reported statistically significantly higher severity for all symptoms (Figure 1A;Supplementary Figure 1, available online). Interestingly, anxiety declined substantially from D1 to D5, which remained low until the end of study at 1 M (Figure 1A and Table 2; *P* < .001). No statistically significant changes were observed for depression (Figure 1A), physical or mental fatigue, activity, or motivation over the course of RT (Table 2;Supplementary Figure 1, available online).

**Table 2.** Changes in symptoms, hematological assessments, and inflammatory markers over time

Variables	Estimated slope	*P* ^a^
General fatigue	0.3613	<.001
Physical fatigue	0.0370	.66
Reduced activity	0.1039	.22
Reduced motivation	−0.0602	.45
Mental fatigue	0.0050	.95
Anxiety	−0.5020	<.001
Depression	−0.0366	.58
Insomnia	−0.3200	.004
CD34^+^	−0.1234	<.001
CD45^+^	−347.9523	<.001
WBC	−0.3295	<.001
Lymphocytes	−0.2017	<.001
RBC	0.0197	.04
Hgb	0.3005	.23
Platelets	−6.5029	<.001
CRP	−189 409.1019	.67
IFN-r	−0.0198	.96
IFN-α2a	−0.0160	.05
IL-10	0.0046	.64
IL-17A	−0.0674	.63
IL-1β	−0.0172	.007
IL-1ra	−6.2429	.04
IL-4	−0.0014	.08
IL-6	−0.0040	.89
IP-10	−11.7488	.34
MCP-1	5.2251	.03
MMP-2	2311.5574	.001
MMP-9	−4926.8319	.27
SDF-1a	5.3105	.63
TGF-β1	−818.7311	<.001
TNF-RII	58.0424	.38
TNF-α	0.0995	.24

a Two-sided *P* values were obtained from F tests in the mixed effects model using D1, D2, D5, and EOT results. CRP = C-reactive protein; Hgb = hemoglobin; RBC = red blood cell; WBC = white blood cell.

Given the reported association of high body mass index (BMI) with worse quality of life in BC patients receiving radiotherapy or chemotherapy (27), BMI data were collected from 77 available case patients and analyzed according to these symptoms. Neither continuous nor dichotomized BMI had statistically significant effects on the trend of general fatigue, insomnia, or mental fatigue (data not shown).

### Changes and Associations Between Symptoms and Hematologic Assessments

CD34^+^, CD45^+^, white blood cell (WBC), lymphocyte, and platelet counts decreased over time, with the lowest levels observed at EOT (Figure 1B and Table 2; Supplementary Table 1, available online; *P* < .001 for all). Patients who received chemotherapy (n = 52) had statistically significantly lower baseline levels of circulating CD34^+^, CD45^+^, WBCs, and lymphocytes than those who were treated with RT alone (Figure 1B;Supplementary Table 3, available online). Longitudinal trends, however, were similar in both groups and were unaffected by RT dose or technique, age, or menopausal status. General fatigue increased and was inversely associated with hemoglobin levels (Table 3; *P* = .02). Increased insomnia was also associated with the reduction of lymphocytes (Table 3; *P* = .04). Additionally, the association between insomnia and fatigue, anxiety, and depression was statistically significant (Supplementary Table 4, available online). No statistically significant associations were found, however, between general fatigue or insomnia with HSCs (Table 3).

**Table 3.** Relationship between symptoms, hematological assessments, and inflammatory biomarkers before and during radiation therapy

Variables	General fatigue		Mental fatigue		Insomnia	
	Estimated slope	*P* ^a^	Estimated slope	*P* ^a^	Estimated slope	*P* ^a^
CD34+	−0.1623	.51	−0.3510	.12	−0.1113	.74
CD45+	0.0000	.70	−0.0001	.36	−0.0001	.38
WBC	−0.0619	.54	−0.0741	.41	−0.1783	.18
Lymphocytes	−0.3777	.14	−0.1891	.40	−0.6663	.04
RBC	−0.6752	.10	−0.6324	.09	−0.1182	.82
Hgb	−0.0326	.02	−0.0160	.21	−0.0163	.39
Platelets	0.0040	.29	0.0010	.77	−0.0051	.31
CRP	0.0000	.60	0.0000	.43	0.0000	<.001
IFN-r	0.0059	.54	0.0122	.15	0.0257	.03
IFN-α2a	0.5052	.24	0.6907	.08	1.2834	.02
IL-10	0.2858	.45	0.3152	.35	0.2325	.63
IL-17A	−0.0241	.36	0.0213	.36	0.0806	.02
IL-1β	0.4223	.53	0.2174	.71	1.2162	.12
IL-1ra	0.0020	.08	0.0002	.83	0.0012	.43
IL-4	1.4726	.72	5.6668	.12	9.6951	.08
IL-6	−0.1025	.44	−0.1063	.37	0.1153	.49
IP-10	0.0007	.03	0.0000	.99	0.0010	.01
MCP-1	0.0011	.44	0.0007	.59	0.0016	.42
MMP-2	0.0000	.88	0.0000	.03	0.0000	.85
MMP-9	0.0000	.87	0.0000	.12	0.0000	.64
SDF-1a	0.0004	.47	−0.0004	.34	0.0004	.51
TGF-β1	0.0000	.56	0.0000	.20	0.0000	.28
TNF-RII	0.0001	.02	0.0000	.38	0.0002	.006
TNF-α	0.0486	.35	0.0169	.71	0.1047	.10

^a^ Two-sided *P* values were obtained from F tests in the mixed effects model using D1 (pretreatment), D2 (second fraction), D5 (fifth fraction), and EOT (end of treatment) results. CRP = C-reactive protein; Hgb = hemoglobin; RBC = red blood cell; WBC = white blood cell.

### Correlation Between Fatigue, Insomnia, and Inflammatory Cytokine Biomarkers During RT

**Figure 2.**
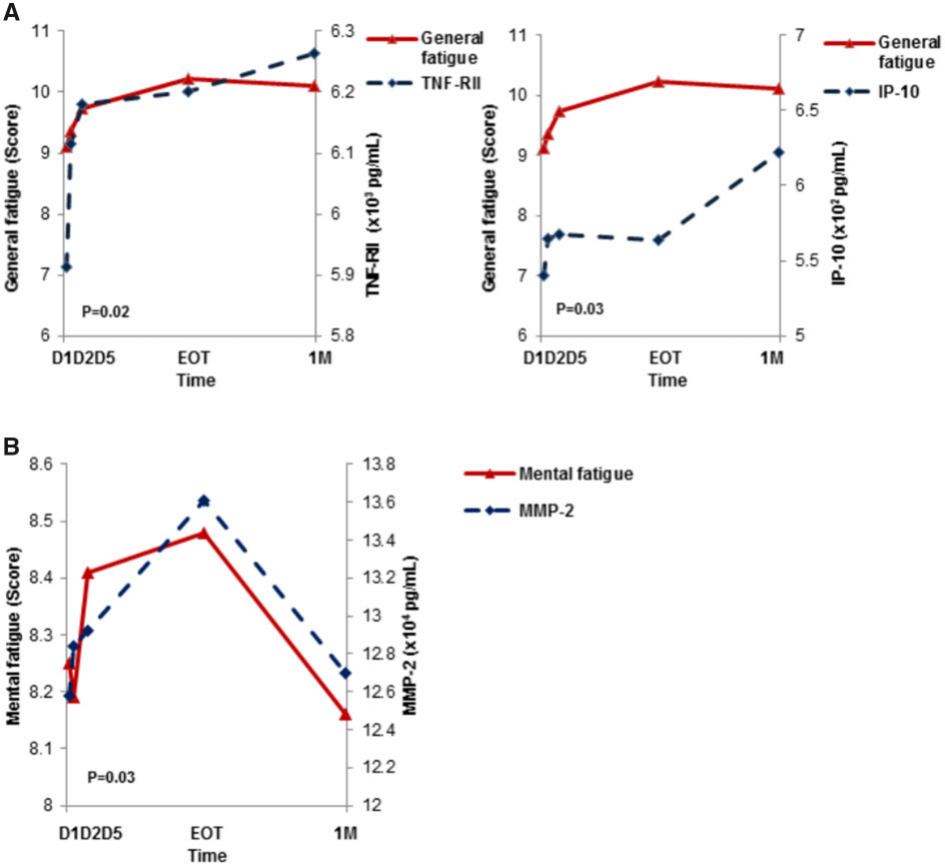
Associations among cytokines with general fatigue and mental fatigue. **A**) General fatigue is associated with TNF-RII and IP-10. Data from the entire cohort of patients (n = 147) demonstrated a positive correlation between TNF-RII (*P* = .02) and IP-10 (*P* = .03) with increasing general fatigue during the course of radiation therapy (RT). **B**) Association of mental fatigue with MMP-2 during the course of RT. MMP-2 is positively associated with mental fatigue during the course of RT for all 147 patients (*P* = .03). 1M = 1 month post-RT; D1 = pretreatment; D2 = second fraction; D5 = fifth fraction; EOT = end of treatment.

The relationship between fatigue, insomnia, and inflammatory biomarker levels during RT was analyzed using the mixed effects model. Changes in IP-10 and TNF-RII levels were associated with worsening of general fatigue (Figure 2A and Table 3; *P* = 0.03 and *P* = .02, respectively), and statistically significant associations were observed between insomnia and CRP, IFN-r, IFN-α2a, IL-17A, IP-10, and TNF-RII (Table 3; *P* < .05 for all). MMP-2 was the only cytokine that was statistically significantly associated with mental fatigue (Figure 2B and Table 3; *P* = .03). None of the other 10 tested biomarkers had any statistically significant association with patient symptoms.

**Figure 3.**
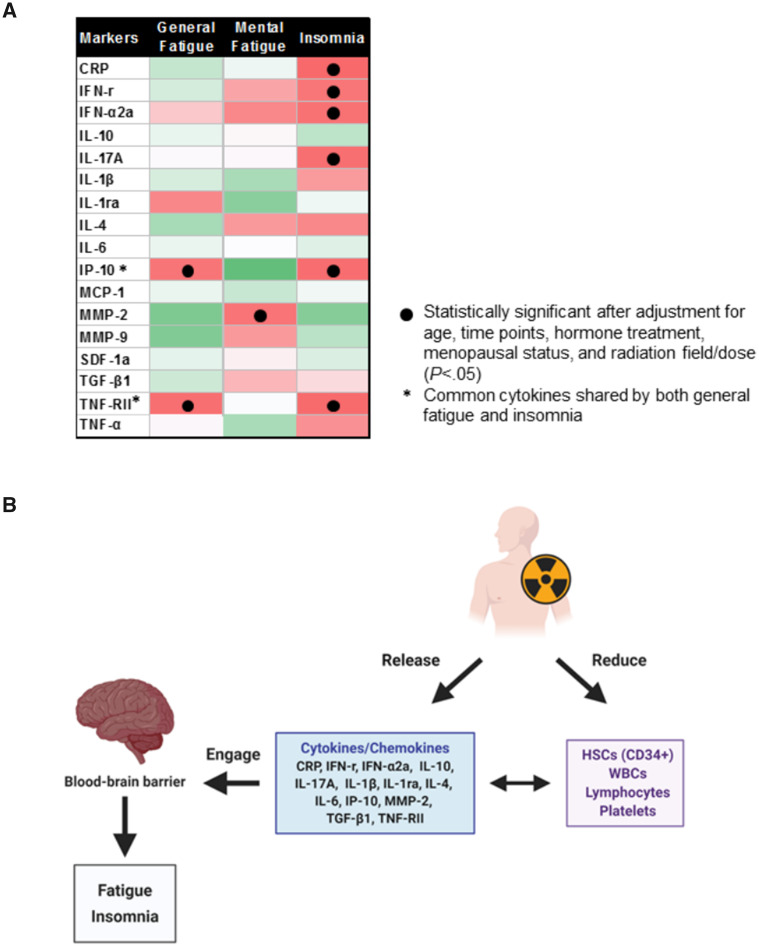
The association of symptoms with inflammatory biomarkers. **A**) Different cytokines are associated with symptoms in RT following adjuvant chemotherapy. Statistically significant associations between cytokines and symptoms are indicated with **black dots** (*P* < .05); cytokines shared by general fatigue and insomnia are denoted with an **asterisk** (*). **Red** represents a positive correlation; **green** represents a negative correlation. **B**) A hypothetical model illustrating the possible mechanisms underlying the development of symptoms in BC patients undergoing adjuvant RT. Local RT induces the release of cytokines/chemokines such as IP-10, TNF-RII, and MMP-2 through a local inflammatory response. The released cytokines engage with transporters in the blood–brain barrier, affecting hypothalamic function, leading to changes in symptoms (e.g. elevated IP-10 and TNF-RII induce general fatigue and insomnia, and MMP-2 is strongly associated with mental fatigue). Local RT also reduces circulating HSCs, WBCs, lymphocytes, and platelets, which in turn appear to strongly associate with cytokines/chemokines such as TGF-β1 and MMP-2. CRP = C-reactive protein; HSC = hematopoietic stem cell; WBC = white blood cell.

After adjusting for age, hormone therapy, radiation fields/doses, and menopausal status, the association between IP-10 and TNF-RII with general fatigue remained statistically significant (*P* < .05 for all). Similar adjustments were performed for insomnia, and the association between insomnia and IP-10 and TNF-RII, as well as CRP, IFN-r, IFN-α2a, and IL-17A also remained statistically significant (Figure 3A and Table 3; *P* < .05 for all). The association between MMP-2 and mental fatigue also remained statistically significant after the adjustment (Figure 3A;*P* < .05).

The correlations observed between changes in circulating HSCs, biomarkers, and cytokines are summarized in Table 4. There was a statistically significant association between the changes in CD34^+^ with IFN-r and TGF-β1. With the exception of MCP-1 and SDF-1a, all other tested cytokines and chemokines showed statistically significant associations with different hematological components (Table 4;Supplementary Tables 1 and 2, available online).

**Table 4.** Relationship between changes in hematological assessments and inflammatory biomarkers before and during radiation therapy

Variables	CD34+		CD45+		WBC		Lymphocytes		RBC		Hgb		Platelets	
	Estimated slope	*P* ^a^	Estimated slope	*P* ^a^	Estimated slope	*P* ^a^	Estimated slope	*P* ^a^	Estimated slope	*P* ^a^	Estimated slope	*P* ^a^	Estimated slope	*P* ^a^
CRP	0.0000	.75	0.0000	<.001	0.0000	<.001	0.0000	1.00	0.0000	.19	0.0000	.02	0.0000	.98
IFN-r	−0.0063	<.001	−3.1739	.44	−0.0021	.59	−0.0059	<.001	−0.0006	.56	−0.0052	.86	−0.0915	.36
IFN-α2a	−0.0874	.23	−186.6709	.31	−0.1035	.58	0.0217	.77	−0.1276	.006	−2.4283	.07	13.7278	.006
IL-10	−0.0926	.14	599.9522	<.001	0.6871	<.001	0.0115	.86	−0.0141	.73	0.2724	.81	1.9561	.64
IL-17A	−0.0021	.64	21.2405	.09	0.0037	.75	−0.0108	.03	0.0007	.80	0.0087	.92	0.2552	.34
IL-1β	−0.0919	.39	−179.9297	.55	−0.1554	.59	−0.2136	.07	−0.0745	.29	−2.3159	.25	−15.1364	.03
IL-1ra	0.0003	.16	3.9516	<.001	0.0036	<.001	0.0005	.008	0.0001	.63	−0.0021	.56	0.0356	.008
IL-4	1.1623	.10	4255.4267	.02	4.5541	.009	0.9211	.18	−0.5546	.21	6.0041	.64	204.2000	<.001
IL-6	−0.0360	.10	259.5381	<.001	0.2680	<.001	0.0210	.41	−0.0012	.94	0.1378	.76	−3.9990	.01
IP-10	−0.0001	.24	−0.0136	.92	−0.0001	.36	−0.0001	.10	−0.0001	.02	−0.0025	.007	0.0153	<.001
MCP-1	0.0002	.44	−0.1076	.87	0.0001	.93	−0.0001	.77	−0.0002	.21	−0.0005	.91	0.0270	.12
MMP-2	0.0000	.05	−0.0046	.06	0.0000	.02	0.0000	.02	0.0000	<.001	−0.0001	<.001	0.0000	.60
MMP-9	0.0000	.14	0.0030	<.001	0.0000	<.001	0.0000	.89	0.0000	.33	0.0000	.44	0.0000	.34
SDF-1a	0.0000	.82	0.3301	.16	0.0002	.30	0.0000	.95	0.0000	.79	−0.0003	.87	0.0058	.34
TGF-β1	0.0000	.008	0.0381	<.001	0.0000	<.001	0.0000	.04	0.0000	.13	0.0001	.21	0.0014	<.001
TNF-RII	0.0000	.13	0.0174	.45	0.0000	.50	0.0000	.09	0.0000	.001	−0.0006	<.001	0.0014	.01
TNF-α	0.0122	.13	16.9024	.45	0.0146	.49	0.0050	.57	−0.0096	.07	−0.3403	.02	0.8576	.11

^a^ Two-sided *P* values were obtained from F tests in the mixed effects model using D1, D2, D5, and EOT results. CRP = C-reactive protein; D1 = pretreatment; D2 = second fraction; D5 = fifth fraction; EOT = end of treatment; Hgb = hemoglobin; RBC = red blood cell; WBC = white blood cell.

Principal Component Analysis supported the above-mentioned findings. The top PC of all the symptom scores covered 66% of the total variance (Supplementary Figure 2, available online). After assessing the association between the top PC and other variables, the results confirmed the statistically significant associations between the symptom profile with changes in HSCs and inflammatory biomarker levels. The detection limits of the multiplexed immunoassays are presented in Supplementary Table 5 (available online).

## Discussion

To our knowledge, this is the first prospective longitudinal study seeking to identify potential common underlying mechanisms of BC RT-related fatigue and other symptoms by measuring cytokines, circulating HSCs, and other hematopoietic biomarkers. As expected, general fatigue progressively increased over the course of RT and was more severe in patients who underwent adjuvant chemotherapy prior to RT. Insomnia improved during the first week but progressively worsened until the end of RT. Interestingly, anxiety levels declined quickly during RT, with no statistically significant changes in depression.

The major findings of this study include the statistically significant associations between the worsening of general fatigue and insomnia with changes in the common cytokines IP-10 and TNF-RII. CD34^+^ (HSCs) and CD45^+^ cells, WBCs, and lymphocytes declined statistically significantly during RT, whereas hemoglobin levels remained unaffected. However, no statistically significant associations were observed between changes in circulating CD34^+^ and CD45^+^ cells with general fatigue or insomnia. Another novel finding was increasing MMP-2 levels being statistically significantly associated with mental fatigue.

Fatigue and insomnia are consistently among the most distressing symptoms experienced by cancer patients undergoing RT and/or chemotherapy. Several studies have identified the clustering of these symptoms (2,7) with a growing body of evidence indicating that pro-inflammatory cytokines may be mediating their development (3,10), supported by our current results (IP-10 and TNF-RII).

Radiation can increase the expression of pro-inflammatory cytokines/chemokines through the activation of NF-kB and STAT prosurvival pathways (28). TNF-α, IL-6, and IL-1β act as “driver” cytokines by either engaging with transporters in the blood–brain barrier or affecting vagal innervation, leading to an imbalance in brain homeostasis (3,29). Although TNF-α, IL-6, and IL-1β were not found to be associated with fatigue or other symptoms in the present study, it is possible that the difference may be due to our longitudinal measurements (vs single point assessments). In contrast, TNF-RII, a downstream marker for TNF activity; IP-10, a chemoattractant; CRP; IFN-r and IFN-α2a, two interferon variants; and interleukin-17A were the most highly induced cytokines in our study, suggesting their possible roles as indicators of a systemic inflammatory response to RT.

This current study also links other cytokines with fatigue and insomnia. Changes in IP-10 were associated with increased fatigue and insomnia. IP-10 is a chemoattractant for activated T cells (30), with important roles in infection, inflammation, and in the central nervous system (31). In rat livers, RT increased both IP-10 and IFN-r (32), with the former also associated with chronic fatigue syndrome (33). The relationship between acute fatigue and IP-10 is a relatively recent observation, with only one previous study reported in patients with acute myeloid leukemia (34), corroborating the observations made in this report.

Our current study strengthens the evidence for an association between sleep disturbance and inflammation. CRP is produced in the liver, and elevated CRP has been associated with sleep deprivation (35). Irradiation-induced differential changes in IL-6 and TGF-β1 also regulate IL-17A release from Th17 cells (36), whereas IL-17A is a potent inducer of CRP expression in hepatocytes (37). The most potent subvariant of interferon alpha (IFN-α) is IFN-α2a (38). In this study, we observed the association between IFN-α2a and insomnia, a finding that has not been previously reported. Therapeutic administration of IFN-α is known to induce fatigue and depression in 30%–70% of patients (39), also causing neuropsychiatric symptoms (40,41). Our current findings provide further evidence suggesting that RT-induced IFN-α2a may play a pivotal role in mediating symptoms of insomnia.

Another novel finding in this study is the relationship between worsening mental fatigue and elevated MMP-2 levels in patients who received RT and adjuvant chemotherapy. The matrix metalloproteinases (MMPs) are a large family of proteases responsible for the degradation of extracellular matrix proteins (42). The balance between MMPs and tissue inhibitors of MMPs is essential for maintaining normal homeostasis, particularly in the CNS (42). Gelatinases MMP-2 and MMP-9 have been implicated in many pathophysiological processes, such as tumor invasion and metastasis (43), but there are limited studies on the role of MMPs in mediating symptoms in patients undergoing adjuvant RT. Overexpression of MMP-2 is associated with radiation-induced damage in various tissues in vitro and in vivo (44). Radiation-induced imbalance between MMP-2 and tissue inhibitors of MMP-2 may disrupt the blood–brain barrier, leading to cognitive impairment in brain tumor patients (45).

The reduction of inflammation may be one strategy to mitigate symptoms during or after treatment for cancer. Although a number of approaches such as exercise, psychosocial, nutritional, and mind–body interventions have shown benefits, elucidating the complexity of inflammatory cytokine networks may provide clues in developing solutions for anti-inflammatory therapies. Currently, infliximab, a TNF antagonist, has been reported to be effective for patients with elevated inflammatory activity (46). Ixekizumab, a monoclonal antibody that selectively targets IL-17A, was shown to reduce depression in approximately 40% of psoriasis patients (47). L-carnitine has been examined in cancer patients for the treatment of fatigue, although this was a negative study (48).

Despite observing several novel associations, there are limitations to the current study. First, we observed relatively low scores for all symptoms in the study population, thereby potentially reducing sensitivity of detection. Second, although a relatively large panel of 17 cytokines was measured, it is highly probable that other cytokines/chemokines may play important roles in mediating the symptoms reported by our patients. Finally, the function of cytokine-cytokine pathways or cytokine-circulating networks is complex, hence an even larger cohort size might be required to further understand these phenomena.

In conclusion, this longitudinal study demonstrated that fatigue and insomnia are symptoms that worsen with RT and adjuvant chemotherapy, and appear to share associations with inflammatory biomarkers. A summary schema of the effects of RT is shown in Figure 3B, but clearly, further studies are required to investigate the complexity of the cytokine networks and their interdependence. These findings will provide new opportunities for developing a mechanism-based strategy to prevent and/or mitigate RT-induced symptoms.

### Funding

This study has been funded in part by the Princess Margaret Cancer Centre Radiation Medicine Program, the Canadian Cancer Society Research Institute (no. 2012–701059), the Canadian Breast Cancer Research Alliance, the Canadian Breast Cancer Foundation, and the Ministry of Health and Long-Term Care.

### Notes

**Role of the funder:** The funders had no role in the design of the study; the collection, analysis, and interpretation of the data; the writing of the manuscript; and the decision to submit the manuscript for publication.

**Conflicts of interest**: The authors have no disclosures.

**Role of the author:** WS, ML, WX, MDM, SVB, KY, and FFL contributed to study design. WS, MM, JW, and DRS performed laboratory experiments. JS, WX, and LSG contributed to statistical and bioinformatics analysis. KH contributed to clinical data collection. WS, SM, LPC, and KY prepared the manuscript with assistance from all the coauthors.

**Acknowledgments:** The authors would like to thank all the study participants and our clinical fellows (Mei Yap, Tim Lymberiou, Ryan Carlson, and Penny Mackenzie), nurses, and clinical trial coordinators (Lea Dungao and Katrina Rey-McIntyre) at the Princess Margaret Cancer Centre.

## Supplementary Material

pkaa037_Supplementary_Data
