# Supplementary material for: Inflammatory Biomarkers, Hematopoietic Stem Cells, and Symptoms in Breast Cancer Patients Undergoing Adjuvant Radiation Therapy
Source: JNCI Cancer Spectr. 2020 May 8;4(5):pkaa037. doi: 10.1093/jncics/pkaa037 (PMC7583146; doi:10.1093/jncics/pkaa037)
Supplement: pkaa037_Supplementary_Data [file pkaa037_supplementary_data.pdf]

Supplementary Methods

HSC measurements

10 µl of fluorescein isothiocyanate (FITC)-conjugated pan-CD45 and phycoerythrin (PE)-conjugated CD34 (class III epitope) antibodies were added to 100 µl of fresh peripheral blood in a Trucount absolute counting tube. After red blood cell lysis, each sample was immediately analyzed using flow cytometry with the single-platform International Society of Hematotherapy and Graft Engineering (ISHAGE) protocol modified for Trucount counting beads. Each sample was counted for a minimum of 100 CD34+ cells with a 15-minute acquisition time. Complete blood counts were obtained using standard hematology laboratory practices (Beckman COULTER® LH 750 Hematology Analyzer, Mississauga, Ontario).

Serum inflammatory biomarker assay

Prior to batch analyses, samples were centrifuged to isolate serum, aliquoted, and frozen at -80°C. A panel of 17 pro-inflammatory and anti-inflammatory cytokines/chemokines were selected for evaluation based on results from our previous studies (17, 18), as well as other studies on inflammatory and cancer-related fatigue, and behavioral symptoms. A customized 10 U-plex biomarker group 1 assay, consisting of IFN-γ, IL-1β, IL-4, IL-6, IL-10, TNF-α, IL-17A, MCP-1, IP-10, and IFN-α2a; two duplex customized MMP-2, MMP-9 and IL-1ra panels, SDF-1a panels, as well as human TNF-RII, TGF-β1 and CRP kits were used (Meso Scale Discovery, Gaithersburg, MD).

Cytokine immunoassays were performed on singly thawed plasma samples. Each sample was run in duplicate using validated commercial Multiplexed Electrochemiluminescence Cytokine Immunoassays from Meso Scale Discovery. Plates were read on a SECTOR® Imager

2400; the detection range varied from 0.5 to 700,000 pg/ml, and all values were expressed as the mean of the measurements.

Supplementary Table 1. Changes of hematologic biomarkers before, during and post-RT (mean (SD); n=147)

Biomarkers	D1 (baseline)	D2	D5	EOT	1M
CD34+ (x10^6/L)	1.40 (0.92)	1.35 (0.82)	1.24 (0.84)	1.00 (0.65)*	1.19 (0.73)
CD45+ (x10^9/L)	5.82 (1.89)	5.86 (1.76)	5.41 (1.68)	4.69 (1.53)*	5.04 (1.64)
WBC (x10^9/L)	6.17 (1.96)	6.16 (1.79)	5.67 (1.69)	5.13 (1.54)*	5.33 (1.68)
Lymphocytes (x10^9/L)	1.85 (0.75)	1.81 (0.66)	1.61 (0.66)	1.15 (0.54)*	1.31 (0.44)
RBC (x10^12/L)	4.27 (0.47)	4.29 (0.44)	4.24 (0.42)	4.36 (0.47)	4.35 (0.41)
Hgb (x10^2g/L)	1.27 (1.7.1)	1.28 (1.32)	1.26 (1.36)	1.29 (1.24)	1.29 (1.44)
Platelets (x10^11/L)	2.19 (5.84)	2.20 (5.64)	2.15 (5.38)	1.88 (5.45)*	1.99 (5.49)

*p<0.001; statistically significant difference between D1 and EOT. All statistical tests were two-sided.

Supplementary Table 2. Changes of inflammatory cytokines before, during and post-RT (mean (SD); n=147)

Cytokines	D1 (baseline)	D2	D5	EOT	1M
CRP (x10^6)(pg/mL)	6.65 (1.52)	6.47 (1.26)	5.16 (0.88)	6.91 (2.02)	5.59 (1.21)
IFN-r (x10^1)(pg/mL)	1.45 (1.97)	1.31 (1.79)	1.42 (1.97)	1.28 (1.06)	1.49 (1.81)
IFN-α2a (pg/mL)	1.25 (0.48)	1.20 (0.45)	1.24 (0.50)	1.06 (0.40)*	1.24 (0.42)
IL-10 (pg/mL)	0.37 (0.51)	0.35 (0.40)	0.37 (0.39)	0.33 (0.21)	0.33 (0.25)
IL-17 (pg/mL)	5.44 (2.99)	5.43 (2.81)	5.70 (7.13)	4.89 (2.77)	5.28 (3.10)
IL-1β (pg/mL)	0.15 (0.30)	0.11 (0.09)	0.10 (0.06)	0.09 (0.06)*	0.11 (0.13)
IL-1ra (x10^2)(pg/mL)	3.28 (2.08)	3.24 (1.86)	3.19 (1.83)	2.81 (1.48)*	3.08 (1.49)
IL-4 (pg/mL)	0.09 (0.05)	0.09 (0.06)	0.09 (0.06)	0.08 (0.06)	0.08 (0.07)
IL-6 (pg/mL)	1.39 (1.08)	1.53 (1.34)	1.39 (1.11)	1.36 (1.19)	1.49 (1.49)
IP-10 (x10^2)(pg/mL)	6.85 (6.43)	7.05 (6.08)	6.77 (5.37)	6.19 (5.51)	6.79 (5.79)
MCP-1 (x10^2)(pg/mL)	3.60 (1.46)	3.66 (1.43)	3.72 (1.43)	3.73 (1.38)*	3.46 (1.33)
MMP-2 (x10^5)(pg/mL)	1.26 (0.32)	1.28 (0.31)	1.29 (0.31)	1.36 (0.36)*	1.27 (0.34)
MMP-9 (x10^5)(pg/mL)	2.13 (2.11)	2.16 (1.82)	1.95 (1.32)	2.14 (1.47)	2.19 (2.01)
SDF-1a (x10^2)(pg/mL)	2.20 (2.84)	2.18 (2.99)	2.38 (4.53)	2.21 (3.41)	2.26 (3.68)
TGF-β1 (x10^4)(pg/mL)	1.79 (0.67)	1.69 (0.62)	1.65 (0.63)	1.47 (0.59)*	1.60 (0.61)
TNF-RII (x10^3)(pg/mL)	6.59 (0.34)	6.77 (0.35)	6.69 (0.34)	6.53 (0.27)	6.47 (0.27)
TNF-α (pg/mL)	3.47 (1.29)	3.54 (1.34)	3.61 (2.03)	3.59 (3.91)	3.43 (1.15)

*p<0.05; statistically significant difference between D1 and EOT. All statistical tests were two-sided.

Supplementary Table 3. . Biomarker changes during, post-RT, without or with adjuvant chemotherapy

Biomarkers	Time	Non-Chemotherapy, n=96 (Mean, SD)	Chemotherapy, n=52 (Mean, SD)
CD34+ (x10^6/L)	D1 (baseline)	1.74 (0.92)	0.79 (0.51)*
	D2	1.65 (0.80)	0.78 (0.49)*
	D5	1.54 (0.85)	0.64 (0.41)*
	EOT	1.21 (0.67)	0.58 (0.32)*
	1M	1.40 (0.73)	0.76 (0.50)*
CD45+ (x10^9/L)	D1 (baseline)	6.48 (1.69)	4.61 (1.64)*
	D2	6.41 (1.61)	4.81 (1.54)*
	D5	6.02 (1.54)	4.21 (1.24)*
	EOT	5.13 (1.49)	3.81 (1.21)*
	1M	5.51 (1.66)	4.08 (1.10)*
WBC (x10^9/L)	D1 (baseline)	6.87 (1.78)	4.91 (1.63)*
	D2	6.75 (1.63)	5.02 (1.53)*
	D5	6.34 (1.48)	4.39 (1.30)*
	EOT	5.61 (1.36)	4.20 (1.44)*
	1M	5.86 (1.69)	4.28 (1.08)*
Lymphocytes (x10^9/L)	D1 (baseline)	2.08 (0.72)	1.42 (0.60)*
	D2	2.01 (0.62)	1.42 (0.53)*
	D5	1.85 (0.64)	1.15 (0.41)*
	EOT	1.26 (0.45)	0.94 (0.62)*
	1M	1.42 (0.45)	1.08 (0.30)*
RBC (x10^12/L)	D1 (baseline)	4.42 (0.44)	3.99 (0.39)*
	D2	4.41 (0.42)	4.05 (0.38)*
	D5	4.35 (0.40)	4.03 (0.38)*
	EOT	4.40 (0.45)	4.29 (0.51)
	1M	4.37 (0.39)	4.31 (0.44)
Hgb (x10^2g/L)	D1 (baseline)	1.30 (0.18)	1.19 (0.12)*
	D2	1.30 (0.12)	1.21 (0.11)*
	D5	1.29 (0.12)	1.20 (0.13)
	EOT	1.30 (0.13)	1.26 (0.09)
	1M	1.32 (0.15)	1.24 (0.09)*
Platelets (x10^11/L)	D1 (baseline)	2.23 (0.56)	2.12 (0.62)
	D2	2.23 (0.57)	2.13 (0.54)
	D5	2.20 (0.55)	2.05 (0.49)
	EOT	1.94 (0.52)	1.75 (0.57)
	1M	2.09 (0.57)	1.81 (0.45)*

* $p<0.01$, statistically significant difference between D1 and EOT. All statistical tests were two-sided.

Supplementary Table 4. Association between insomnia and other measurements

	Statistic	General fatigue	Physical fatigue	Reduced activity	Reduced motivation	Mental fatigue	Anxiety	Depression
Insomnia	R	0.6237	0.577	0.5793	0.5304	0.5339	0.5615	0.6366
	p-value	<0.001	<0.001	<0.001	<0.001	<0.001	<0.001	<0.001

The association between insomnia and all other symptom measures is statistically significant.

Supplementary Table 5. Lower and upper limits of detection (LLOD and ULOD, respectively) for measured inflammatory biomarkers in serum samples (pg/mL)

Biomarkers	LLOD(pg/ml)	ULOD(pg/ml)	In detection Range(%) (mean)	Intra CVs (mean)	Inter CVs (mean)
CRP	12.480	195000.000	99.902	4.628	3.500
IFN-r	4.150	17000.000	99.940	6.916	7.946
IFN-α2a	10.352	42400.000	93.440	13.912	5.485
IL-10	0.907	3716.000	94.303	12.042	6.553
IL-17A	5.703	23360.000	95.803	11.660	6.757
IL-1β	0.933	3820.000	72.605	27.914	4.376
IL-1ra	24.414	100000.000	100.000	3.792	7.558
IL-4	0.504	2064.000	76.904	24.808	7.373
IL-6	0.483	1980.000	96.590	10.028	5.931
IP-10	1.465	6000.000	99.778	4.182	9.752
MCP-1	1.602	6560.000	99.928	3.400	5.012
MMP-2	122.070	500000.000	99.864	3.106	12.881
MMP-9	122.070	500000.000	97.990	2.688	6.091
SDF-1a	9.766	40000.000	87.022	15.684	16.274
TGF-β1	0.100	100000.000	99.726	13.250	15.784
TNF-RII	2.441	10000.000	100.000	3.648	2.787
TNF-α	0.892	3652.000	99.928	3.884	5.501

LLOD: The lower limit of detection
ULOD: The upper limit of detection
Intra CVs: intra-assay coefficient of variance
Inter CVs: inter-assay coefficient of variance

Supplementary Figure 1

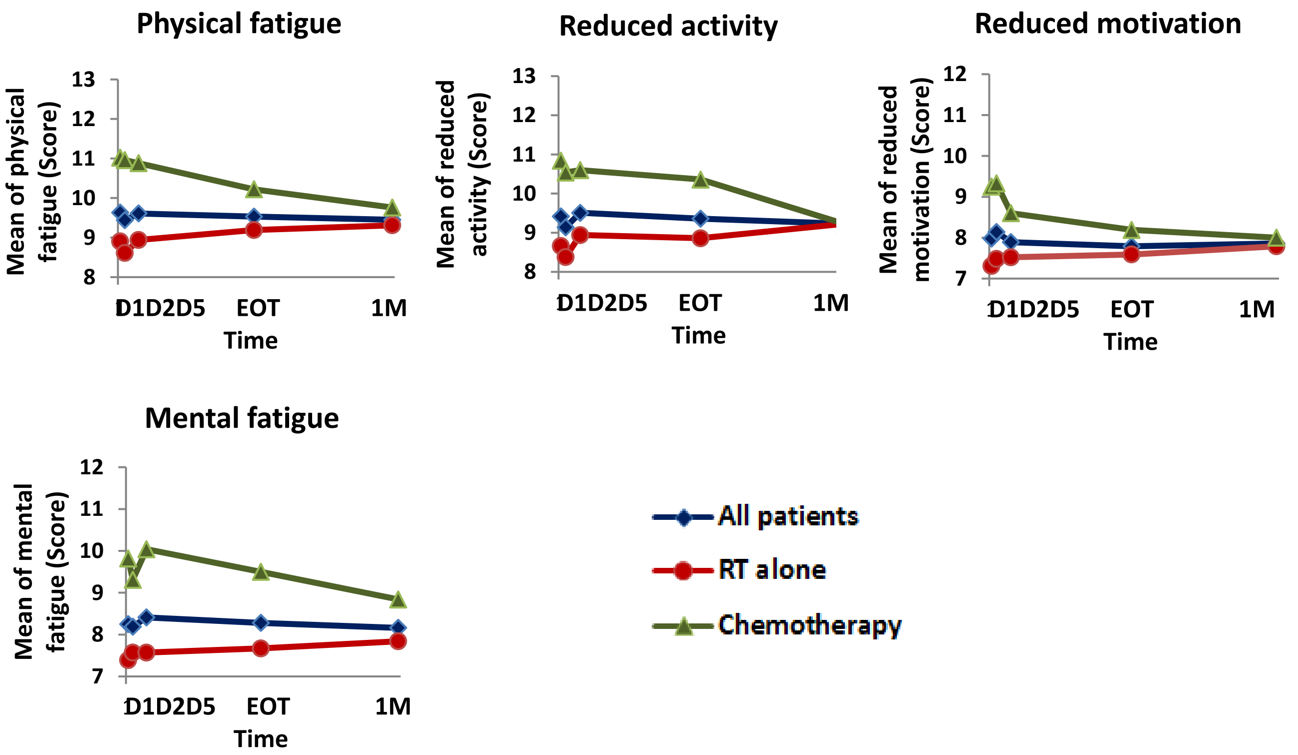

Change in symptoms over time. The average severity of each component score are plotted throughout the duration of RT and 1-month post-RT for the entire cohort (n=147), and then divided as a function of RT only (n=95), or adjuvant chemotherapy (n=52). Overall, there were no significant changes for physical fatigue, reduced activity, reduced motivation, or mental fatigue throughout this time period.

Supplementary Figure 2

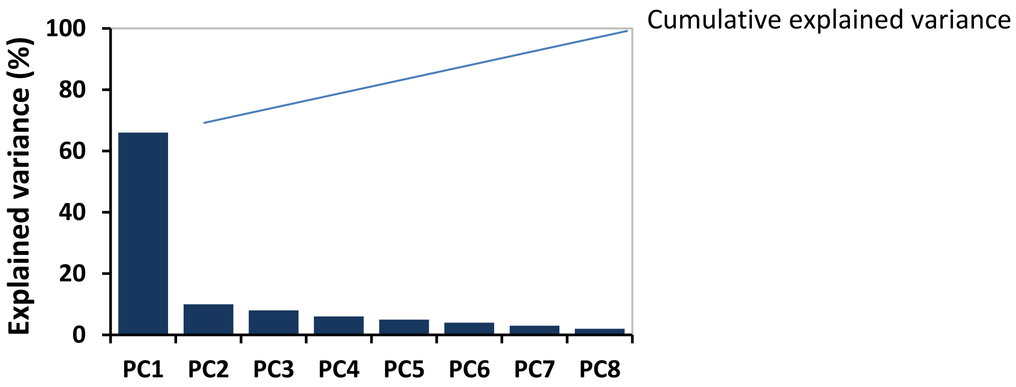

Principal component analysis (PCA) for fatigue score variance. The majority of the total variance of fatigue scores was calculated at PC1 and PC2. The major principal component (PC1) for fatigue scores covered ~66% of variance. Pearson partial correlation coefficients analysis was applied to assess the association between the top PC and other variables. The correlation between the top PCs with cytokines and biomarkers were consistent with the single score assessment.
